# A TATA binding protein regulatory network that governs transcription complex assembly

**DOI:** 10.1186/gb-2007-8-4-r46

**Published:** 2007-04-02

**Authors:** Kathryn L Huisinga, B Franklin Pugh

**Affiliations:** 1 Center for Gene Regulation, Department of Biochemistry and Molecular Biology, The Pennsylvania State University, University Park, PA 16802, USA; 2 Department of Biology, Washington University, Saint Louis, MO 63130, USA

## Abstract

**Background:**

Eukaryotic genes are controlled by proteins that assemble stepwise into a transcription complex. How the individual biochemically defined assembly steps are coordinated and applied throughout a genome is largely unknown. Here, we model and experimentally test a portion of the assembly process involving the regulation of the TATA binding protein (TBP) throughout the yeast genome.

**Results:**

Biochemical knowledge was used to formulate a series of coupled TBP regulatory reactions involving TFIID, SAGA, NC2, Mot1, and promoter DNA. The reactions were then linked to basic segments of the transcription cycle and modeled computationally. A single framework was employed, allowing the contribution of specific steps to vary from gene to gene. Promoter binding and transcriptional output were measured genome-wide using ChIP-chip and expression microarray assays. Mutagenesis was used to test the framework by shutting down specific parts of the network.

**Conclusion:**

The model accounts for the regulation of TBP at most transcriptionally active promoters and provides a conceptual tool for interpreting genome-wide data sets. The findings further demonstrate the interconnections of TBP regulation on a genome-wide scale.

## Abstract 2

A portion of the assembly process involving the regulation of the TATA binding protein (TBP) throughout the yeast genome is modeled and experimentally tested.

## Background

The model eukaryotic cell *Saccharomyces cerevisiae *runs its life with approximately 5,700 genes [1,2]. In any given environment, each gene is expressed at a level that allows the cell to function optimally in that environment. Most genes are lowly expressed and relatively few are highly expressed, which characterizes two ends of an expression continuum [3].

Several general regulatory features dictate the expression levels of every gene [4-6]. First, promoter regions are packaged into chromatin, which regulates promoter accessibility. Second, sequence-specific DNA binding proteins orchestrate the remodeling of chromatin and the recruitment of the transcription machinery. Third, general transcription initiation factors (GTFs) such as TFIIA, -B, -D, -E, -F, -H and RNA polymerase II (pol II) assemble into a transcription pre-initiation complex (PIC). Fourth, pol II and associated elongation factors produce an RNA transcript. Each level of regulation involves many proteins.

Since cells follow the laws of chemistry, the hundreds of proteins regulating RNA production at thousands of genes will require millions of coupled reaction steps. Defining these steps has been a longstanding and continuing effort in transcription biochemistry. A major challenge is piecing together individual steps, defined in isolation, into a biochemical gene regulatory network that describes the totality of a gene expression program *in vivo*. Such a network allows aspects of gene regulatory programs to be modeled computationally, providing a guide for conceptualizing and predicting the complex interplay of regulatory proteins.

The biochemical networks described here differ from previously described genetic networks [7,8]. The latter are typically DNA-centered and describe the spatial and temporal aspects of organismal development as a consequence of an unfolding cascade of chronological gene expression events that control downstream events. Here, biochemical networks are protein-centered, describing transcriptional control in terms of differential equations governing the dynamic interplay among proteins and promoter DNA. The two are related in that biochemical networks drive genetic networks. However, no biochemical model of a global gene regulation network currently exists, and no paradigm exists by which a biochemical model can be tested on a genome-wide scale.

Towards this goal, we have constructed a prototype model that describes one section of the global network in terms of a composite of well-defined biochemical interactions that regulate the function of the TATA binding protein (TBP). From this model, we formally define a reaction mechanism involving interactions among TBP, its regulators, and promoter DNA, ultimately culminating in PIC assembly and RNA production. This formulation is analogous to a mechanism describing a series of coupled enzymatic reactions and, thus, can be computed using a software simulator of enzymatic reactions. The simulator reports steady-state levels of intermediates in PIC assembly and the amount of RNA produced. Perturbations to the network are modeled computationally, and tested experimentally via genetic mutations in the network. This work provides an initial framework for developing and testing biochemically based transcriptional regulatory networks that govern PIC assembly and RNA output, and a means for understanding their design logic.

## Results and discussion

### A model of the TBP regulatory network

The building block for modeling our global biochemical gene regulatory network is an elementary reaction step such as:

P_x _+ D_y _→ P_x_D_y_

where a protein (P) whose identity is 'x' (for example, TBP) binds to DNA (D) located in the promoter of gene 'y' to form a protein-DNA complex (P_x_D_y_), as demonstrated previously [9-11]. The forward flux of P and D through the reaction is governed by a gene-specific flux constant k_1_. P_x_D_y _may be coupled to a second reaction step such as:

P_x_D_y _+ P_z _→ P_x_D_y_P_z_

exemplified by a TBP·DNA complex binding the TBP regulator called negative cofactor 2 (NC2). Examples of other types of reaction steps include protein-protein assembly, protein-DNA disassembly, rearrangements within a complex, and chemical catalysis.

In principle, hundreds of different transcription regulatory proteins can act upon each other and upon thousands of genes, giving a nearly infinite combination of potential reaction steps. In reality, biologically relevant interactions have specificity, which keeps the number of reaction steps to a finite but nevertheless large number. In constructing a biochemical gene regulatory network, we employed only reaction steps that have strong experimental support. TBP regulatory mechanisms are among the best characterized eukaryotic gene regulatory systems [12-14], and thus are ideally suited for integrative modeling studies.

**Figure 1:**
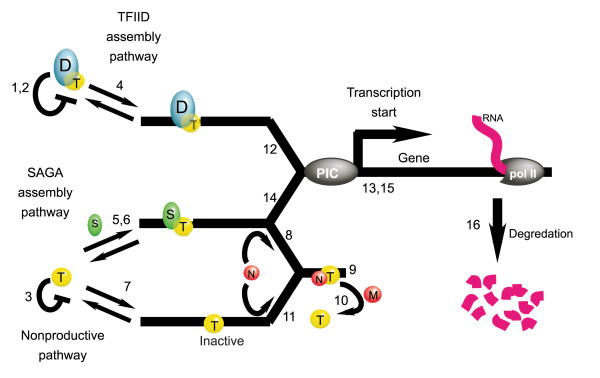
An integrated prototype of the TBP regulatory network. Three parallel assembly pathways proceed temporally from left to right along line segments that represent promoter DNA. Two pathways, directed by the multisubunit TFIID complex ('D', in cyan) and the compositionally related SAGA complex ('S', in green) lead to productive pre-initiation complex (PIC) assembly, which goes on to produce RNA. The RNA ultimately is degraded leading to a steady-state balance between production and degradation. In this model, TBP ('T', in yellow) resides as a self-inhibited dimer when not bound to DNA. Dissociation into monomers is required for DNA binding. The TAND domain of TFIID's TAF1 subunit further inhibits TBP binding to DNA along the TFIID pathway. The third pathway loads TBP onto promoter DNA in a nonproductive manner, and interferes with PIC assembly unless dissociated by the combined action of NC2 and Mot1 ('N' and 'M' in red) [24]. NC2 and Mot1 also dissociate TBP loaded via the SAGA pathway. While Mot1 dissociates TBP in the absence of NC2 *in vitro*, it appears to be linked to NC2 function *in vivo *(Figure S1 in Additional data file 1). Numbers correspond to steps defined in Figure 2a. The presence of chromatin and general transcription regulators is implicit and not shown because their contributions to PIC assembly are not being tested here.

Figure 1 illustrates the prototype TBP regulatory network upon which our studies are based. The model attempts to assimilate a variety of individual TBP regulatory mechanisms into a common regulatory network that is potentially applicable to all genes. This represents the first description of an integrated TBP regulatory mechanism. The model serves as a visual framework for interpreting *in vivo *promoter occupancy and gene expression data. While parts of the model could be wrong or incomplete, it serves as a useful starting point to evaluate whether potential gene regulatory mechanisms defined *in vitro *with purified components can be integrated into a network of coupled reactions that account for transcription factor occupancy and gene expression profiles *in vivo *on a genome-wide scale. The goal here is to take a step towards bridging a myriad of *in vitro *biochemical mechanisms with genome-scale *in vivo *regulatory processes, rather than to establish a rigorous mathematical model for regulatory networks.

In this model, TBP resides as a self-inhibited dimer when not bound to DNA (segments 2 and 3 in Figure 1) [9,15]. Dissociation into monomers, as directed by promoter-specific regulators, is required for DNA binding. When TBP resides in the multisubunit TFIID complex, it may also engage in interactions with other TFIID subunits such as the TBP-associated Factor 1 (TAF1) amino-teminal domain (TAND) (segment 1 in Figure 1) [16,17]. TAND has the potential to act negatively by blocking TBP's DNA binding surface, and positively by tethering TBP to TFIID. Although not shown in the model, promoter-bound regulators modulate the assembly of TFIID-TBP at promoters [18,19], giving rise to promoter-specific control of the network.

The model shows TBP assembling onto promoter regions via three possible pathways. One pathway involves TFIID (segment 4) and a second involves the Spt-Ada-Gcn5-Acetyltransferase complex termed SAGA (segments 5,6). Both lead to formation of a PIC containing TBP, pol II, and many other transcription proteins (segments 12 and14). The PIC produces a transcribing pol II from which RNA is made (segments 13 and 15). The third pathway, outlined in more detail below, is a nonproductive one.

TFIID and SAGA are compositionally and functionally related complexes [20,21]. In principle, a given promoter can utilize either the TFIID or SAGA pathway [22]. The SAGA pathway is tailored towards TATA-containing promoters, whereas the TFIID pathway plays a greater role at TATA-less promoters [23].

Inhibiting the SAGA pathway (but not the TFIID pathway) are two negative regulators of TBP, termed NC2 and Mot1 (Figure 1, segments 8, 9, and 10) [22,24]. NC2 binds to a TBP-DNA complex and blocks PIC assembly [25,26]. Mot1 uses the energy of ATP hydrolysis to dissociate TBP from DNA [11,27,28]. Mot1 can also dissociate TBP from DNA in the presence of NC2 [24], and NC2 stimulates TBP-Mot1 interactions [29]. Since the genome-wide gene expression profile of an NC2 mutant is very similar to the expression profiles of Mot1 mutants (Figure S1 of Additional data file 1) [30,31], we make the simplifying assumption that the two largely work together. Consistent with the notion that Mot1 dissociates TBP-NC2 complexes, Mot1 mutants result in the accumulation of TBP-NC2 complexes *in vivo *[32]. However, we do not exclude the possibility that Mot1 might act in the absence of NC2 at some promoters.

The third TBP assembly pathway is nonproductive (Figure 1, segment 7), as has been proposed by Dasgupta *et al*. [33]. This nonproductive pathway has the potential to compete with the two productive pathways and inhibit transcription. At genes where this nonproductive pathway limits transcriptional output, removal of the inactive TBP by the coordinate action of NC2 and Mot1 (Figure 1, segments 11, 9, and 10) would, therefore, result in positive regulation [33]. At genes where the SAGA pathway dominates, NC2 and Mot1 would act negatively. Thus, the model has the capacity to account for positive and negative regulation by NC2 and Mot1, both of which have been reported for these proteins [30,31]. The model is limited in that it does not include other types of regulatory functions that SAGA (Spt3), Mot1, or other network components might be involved in [34], but for which there is no direct biochemical support.

Biological systems are a balance of assembly and disassembly processes that typically move along different pathways. Thus, PIC disassembly and RNA degradation do not proceed by an exact reversal of PIC assembly and transcription. Nevertheless, proteins and RNA are recycled, allowing gene expression to reach a steady-state in a stable environment. Therefore, our modeled biochemical gene regulatory network is designed to reach steady-state, where the rate of PIC assembly and RNA production equals the rate of PIC disassembly and RNA degradation (Figure 1, segment 16).

Within the cell, the TBP regulatory network is embedded within the larger global gene regulatory network involving hundreds of functionally diverse proteins, such as activators, chromatin remodeling complexes, mediator, and general transcription factors. They are not an explicit part of this modeling study (and thus not shown in Figure 1) because they are assumed to make contributions to PIC assembly and RNA output that are approximately equivalent in the mutant and wild-type strains used here. For example, a TBP mutation that impairs TBP-NC2 interactions is assumed to have a negligible effect on other types of interactions within chromatin and the transcription machinery.

### Computational simulation of a TBP regulatory network

The core mechanism, as defined by the model, is tailored to specific genes by adjusting the flux constants. For example, the flux constant governing the association of TBP with one promoter may be different to the flux constant governing TBP association with a different promoter as dictated by promoter-bound activators [35-37]. In our modeling studies, promoter-specific factors such as bound regulatory proteins and chromatin structure are not individually defined because their individual contributions are not being tested here. The flux constants reflect their combined contributions.

**Figure 2:**
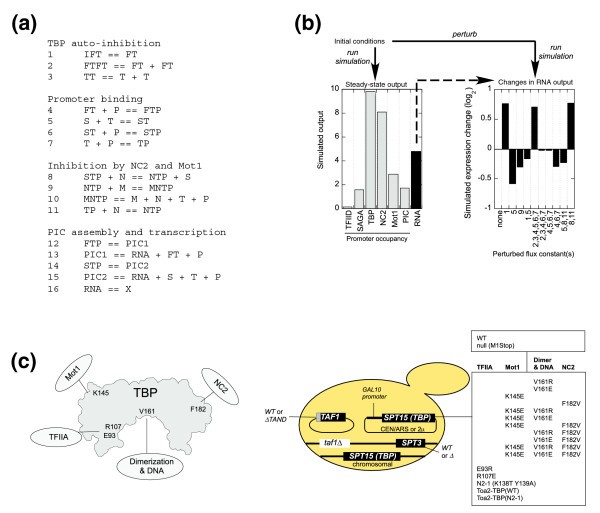
Simulation of the TBP regulatory network. **(a) **The reaction mechanism as entered into the KinTekSim program. Each numbered line represents a reaction segment governing TBP regulation and PIC assembly shown in Figure 1. The '= =' denotes forward and reverse reactions governed by 16 forward and 16 reverse "flux constants". See Additional data file 1 for definitions of terms. **(b) **Simulated output. The simulator was initiated with a set of values for species concentrations and flux constants governing each numbered reaction segment in (a) (in this example we used values from cluster 5 described below). The simulation was run to steady-state. The reported steady-state concentrations of the indicated protein-promoter complexes (gray bars, left graph) and RNA (black bar, left graph) are plotted as output in arbitrary units. In the right bar graph, the indicated flux constants governing the reaction segments defined in panel (a) were reduced to mimic the physiological effect of a mutation. Changes in RNA output resulting from the perturbation are plotted on a log_2 _scale, as is common for expression profiling. **(c) **Experimental design for probing the TBP regulatory network. The left illustration is an outline of TBP with the approximate location of amino acids targeted for mutagenesis, and the interactions that are affected. The right illustration describes the strain design. Four basic strains derived from Y13.2 were constructed that contained or lacked the TAF1 TAND domain (TFIID pathway) and contained or lacked *SPT3 *(SAGA pathway). A plasmid expressing the indicated HA-tagged TBP mutants under the control of the *GAL10 *promoter was introduced into each strain, with the exception that the TFIIA-interaction mutants were introduced only into the WT and *taf1*(*ΔTAND*) strains. TBP plasmids in the *spt3Δ *strains utilized the 2 micron origin. Since full galactose induction requires SAGA (Spt3), it was necessary to employ the 2 micron high copy plasmid and a longer induction period in the *spt3Δ *strains to achieve TBP expression levels comparable to those in wild-type *SPT3 *strains. These procedural modifications did not alter the experimental outcomes, except to provide quantitatively more robust effects (data not shown). Evidence that the TBP mutations affect the targeted interactions is provided in section 2 of Additional data file 1.

The model illustrated in Figure 1 was converted into a series of explicitly defined reaction segments (Figure 2a) that were suitable for computational modeling using the freely available KinTekSim simulator [38]. KinTekSim is a derivative of KINSIM, which is used to model enzyme reaction mechanisms [39]. For any set of similarly behaving genes, the core mechanism contains 16 segments governed by 16 forward and reverse 'flux constants'. Although written as explicit reaction steps, these segments are intended to approximate relevant portions of the modeled network that have been biochemically demonstrated. The flux constants provide a means for governing the relative distribution of components in the pathway when the simulation has reached steady-state.

The distinct arrangement of promoter elements and bound proteins ranging from activators to chromatin is expected to control the flow of components through every step in the network (that is, flux constants) in a way that is unique to every gene or set of co-regulated genes. This includes segments of the mechanism that are not explicitly marked in Figure 1 as involving promoter-bound regulators (for example, segments 1-3).

One challenge is fitting the model to experimental data within the constraints imposed by the experimental system. We required our computational model to report outputs that had experimental counterparts such as promoter occupancy from ChIP-chip assays or mRNA levels. Experimentally, such absolute levels obtained on a genome-wide scale are inherently noisy. Noise is reduced substantially by measuring changes in occupancy or changes in mRNA levels. Therefore, we set up our model to report both steady-state and changes in steady-state levels when a portion of the network is shut down. Computationally, we shut down a portion of the network by reducing the flux constant governing that portion of the network. Mutagenesis is used to achieve the physiological counterpart (see below). Further noise reduction is achieved by combining data within a set of similarly behaving genes so that hundreds of measurements contribute to the modeled data. Combining data also reduces the computational complexity from approximately 5,700 core mechanisms for approximately 5,700 genes to a manageable small number.

Figure 2b (left panel) demonstrates how initialization of the flux parameters governing one gene (or group of co-regulated genes) leads the simulator to produce promoter occupancy intermediates and RNA outputs (in arbitrary units) that have experimental counterparts measurable with ChIP-chip and expression microarrays. By altering flux constants that govern the progress of the coupled reactions, we control the steady-state levels of these components.

Changing the values of the flux constants, mimicking either gene control or genetic mutations that impair an interaction, leads to changes in factor occupancy and RNA output. Figure 2b (right panel) plots simulated changes in RNA output (log_2 _scale) when one or more flux values are altered. In some cases the impact is substantial, while in others the impact is minimal, reflecting the extent to which a TBP regulatory interaction contributes to the expression of that gene.

Using this approach, we tested the simulator and the model by creating mutant yeast strains having single and multiple defects in biochemically defined TBP regulatory interactions (Figure 2c, and section 1 of Additional data file 1). These mutations provide the physiological counterpart to perturbations of the computational network. The experimental design has several constraints. First, we wanted to minimize potential indirect effects from physiological adjustments arising from long-term exposure to the mutations. Therefore, we placed all TBP mutants under control of the *GAL10 *promoter and analyzed gene expression changes throughout the genome after induction of TBP with galactose. TBP levels pre- and post-induction along with endogenous wild-type TBP are shown in Additional data file 1 (section 1 of Figure S2). The reference state in all experiments is a strain where a 'null' TBP is induced. Induction of wild-type TBP had no significant effect on the system, indicating that the measured effects are unlikely to be inherent in the experimental design and thus are likely to be caused by the mutations.

Second, because TBP is essential for cell growth it was necessary to include endogenous wild-type TBP in all strains. Competition between the mutant and wild-type TBP is likely to diminish, but not eliminate, the impact of the mutants. Indeed many mutants displayed dominant growth phenotypes (Figure S3 of Additional data file 1). Validating the use of TBP mutants in the context of wild-type TBP, a nonlethal TBP mutation generates similar genome-wide expression profiles in the presence or absence of endogenous wild-type TBP (Figure S4a, subpanel 4, of Additional data file 1). Therefore, the presence of wild-type TBP in the background is not expected to substantially impact the correlations between simulated and actual data.

It was not feasible to eliminate all TBP regulatory interactions via mutagenesis of TBP. First, since TBP's interaction surface with SAGA remains ill-defined, this interaction was eliminated by deleting *SPT3*, which encodes a TBP regulatory subunit of SAGA [40,41]. Second, TBP mutations that impair interactions with TFIID do not generate the same phenotype as deletion of TFIID's main TBP interaction domain, the TAF1 TAND domain [16,17,42-45]. Since this region of TBP interacts with other factors as well, we opted to delete the TAND domain as a means to diminish TBP-TFIID interactions. Third, we cannot be certain that the mutations affect only the interactions being modeled, and thus is a caveat of any *in vivo *analysis. However, the mutated residues employed in our study have been biochemically characterized for their defective interactions [27,42,46-48]. In the context of our study, we sought to provide further evidence on a genome-wide scale that the mutants were generally having the intended effects *in vivo*. These validation studies are presented in section 2 of Additional data file 1, and support the assertion that the mutants are defective in the modeled interactions.

### Identifying genes that are sensitive to the TBP regulatory network

Changes in mRNA output at more than 99% of all known *S*. *cerevisiae *genes were measured in 63 wild-type or mutant strains (Additional data file 2). Of these, 2,903 genes met stringent filtering criteria for changes in gene expression (see Materials and methods) and thus were chosen for the modeling studies. Most of the remaining approximately 3,000 genes were not significantly expressed (cluster 0 in Table 1, entry 12), which may be due to repressive mechanisms (for example, repressors, chromatin, and so on) that lie outside of the TBP regulatory network. Their lack of expression can be modeled by setting the forward flux constants to zero. The TBP regulatory network may be applicable to these genes under conditions where they are expressed.

**Figure 3:**
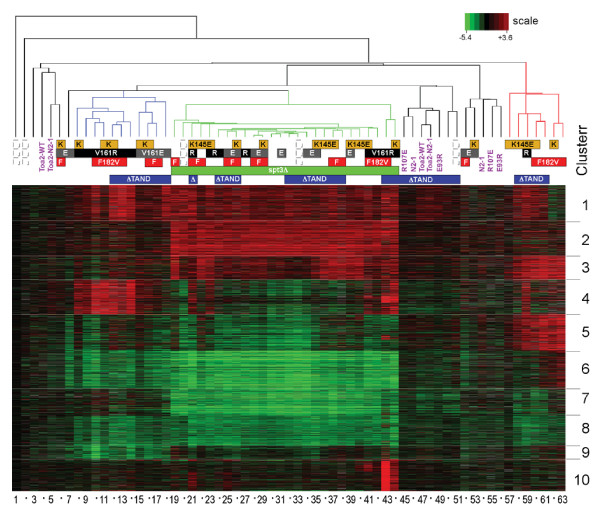
Genome-wide changes in expression upon perturbation of the TBP regulatory network. We grouped 2,903 ORFs (rows) that changed expression by at least 1.5-fold in at least one of the 63 conditions (columns) examined into 10 clusters using the K-means algorithm [63]. Clusters 1-10 contained the following number of ORFs, respectively: 338, 333, 225, 330, 344, 359, 259, 282, 127, 306; 3,323 ORFs did not meet the filtering criteria. Changes are relative to a galactose-induced null TBP in a wild-type TBP background. Red, green, and black denote increased, decreased, and no change in gene expression. Gray denotes no data. Color intensity reflects the magnitude of change on a log_2 _scale. Columns were arranged by hierarchical clustering [63]. Color boxes above each column indicate the relevant strain genotypes. All mutations are located on TBP except where designated '*spt3Δ*', and 'ΔTAND', which signify deletion of *SPT3 *and the *TAF1 *TAND domain. Boxes with dashed outlines indicate null TBP. All strains harbor a chromosomal copy of the endogenous wild-type TBP gene (*SPT15*).

Individually applying the core mechanism to each of the 2,903 filtered genes was not practical. Instead we sought to identify major themes in the network. This was achieved by combining data from similarly behaving genes. Data consolidation enhanced the robustness of modeling since any one consolidated value is derived from as many as several hundred data points that were not appreciably different from each other (20% average standard deviation; see Additional data file 2). We used a K-means algorithm to assist in clustering the data, and identified ten as the maximum number of nonredundant clusters (Figure 3). Since all genome-wide measurements naturally fall into a continuum of values rather than well-partitioned clusters, the choice of a cluster number is necessarily subjective. Partitioning the data into more clusters generated increasingly subtle quantitative differences rather than major qualitatively distinct patterns. Genes within a cluster were consolidated by calculating median changes in gene expression for each mutant in each cluster.

Of the ten clusters, six (clusters 3-6 and 8-9) were deemed appropriate for modeling studies. The properties of the remaining four clusters (clusters 1, 2, 7 and 10) suggested that they arose from indirect effects. The complex interconnectivity of biological systems ensures that some changes in gene expression in response to a genetic perturbation will necessarily cause other genes to change in expression, with the latter being largely an indirect consequence of the primary perturbation. Therefore, indirect responders are not accurate metrics of a perturbed network. Detailed justifications for their exclusion are presented in section 3 of Additional data file 1, which is briefly summarized here.

Cluster 1 values were inversely correlated with cluster 8 values (Figure S5 in Additional data file 1). The behavior of cluster 8 could be accounted for by well known biochemical interactions that TBP engages in. Accounting for the pattern in cluster 1 as a direct effect of TBP required TBP to possess biochemical activities that are the opposite of its known activities. Since there is no basis for such supposition, we interpret the inverse relationship between clusters 1 and 8 as cluster 8 genes being the predominant inhibitor of cluster 1 genes, rather than TBP reversing its activity. Strikingly, cluster 8 genes encode predominantly ribosomal proteins whereas cluster 1 genes are involved predominantly in ribosomal biogenesis (Table 1, rows 3 and 4), suggesting that ribosomal protein genes or their products down-regulate ribosomal biogenesis genes. In support of this notion, cluster 1 genes are highly enriched with genes that are up-regulated when ribosomal protein genes are deleted (Table 1, row 26).

Cluster 2 genes were up-regulated only in *spt3Δ *strains. However, ChIP-chip occupancy measurements indicated that Spt3 was not present at these genes in wild-type strains (data not shown), suggesting that the changes in gene expression are likely to be an indirect consequence of physiological adjustments to the constitutive absence of Spt3. Cluster 7 genes were down-regulated only in *spt3Δ *strains. Cluster 7 was enriched with genes involved in Ty transposition (Table 1, row 10), which is particularly Spt3 dependent [49]. Because of the possibility of unique regulation of Ty elements and the potential for long-term physiological adjustments to the constitutive *spt3 *deletion, cluster 7 genes were not modeled.

Almost all of cluster 10 genes were restricted to chromosomes XI and XII (Figure S6 in Additional data file 1), suggesting that the highly mutated strains that demarcate this cluster underwent duplications of chromosomes XI and XII. Therefore, the apparent increase in expression is likely due to an increase in gene copy number, and thus was excluded from the analysis.

### Testing the TBP regulatory network through genome-wide occupancy assays and expression profiling

Clusters 3-6 and 8-9 represent six classes of genes that display dependencies on different parts of the TBP regulatory network. The challenge is to determine whether the flux constants governing the network can be fit with values that recapitulate these dependencies. A proper fit requires a match between actual changes in gene expression, when a section of the network is genetically impaired, and changes that are simulated when the relevant flux constant(s) is/are altered in value. An important caveat here is that a good fit does not necessarily indicate that the model is correct since there are more degrees of freedom than there are observations to constrain the model. Nevertheless, the modeling provides a tool for integrating and interpreting massive genomic data sets into a plausible conceptual model.

**Figure 4:**
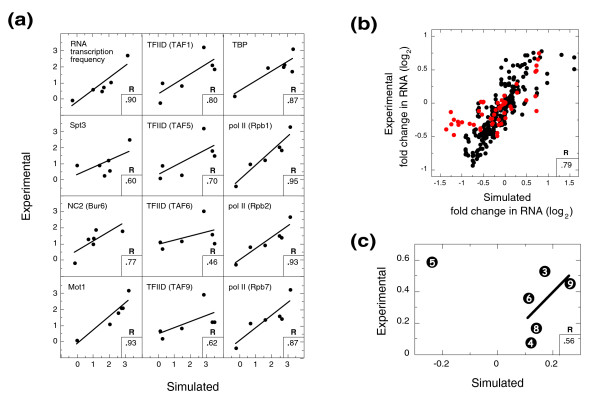
Testing the TBP regulatory network through genome-wide occupancy and expression profiling. **(a) **Correlations in RNA and factor occupancy levels, comparing simulated and experimental data. The flux constants governing the core reaction mechanism in Figure 2a were initialized to values specific to clusters 3, 4, 5, 6, 8, and 9 (Additional data file 3). The simulation was run to steady-state. RNA output and the levels of promoter-bound SAGA (Spt3), NC2 (Bur6), Mot1, TFIID (TAF1, TAF5, TAF6, TAF9), and PIC (Rpb1, Rpb2, Rpb7) were reported. These values were then compared to experimentally measured levels of RNA (transcription frequency as defined by [3], and the indicated factor occupancy measured by ChIP-chip [50,52]). Experimental values are median values for each of the six clusters (hence six data points). The x and y axes are on a linear scale. The units for each plot are relative, and thus have no intrinsic meaning except to provide a linear scale demarcating the relative concentration of RNA or promoter-bound species. Correlation coefficients (R) are indicated in the lower right corner of each plot. **(b) **Correlations between simulated and actual changes in RNA output when the TBP regulatory network is perturbed. The scatter plot reflects changes in actual (from Figure 3) and simulated RNA output relative to a wild-type cell. Median values for each mutant in each of the six clusters are plotted. Cluster 5 data are shown in red. The x and y axes are plotted on a linear scale, with values log_2_-transformed. The correlation coefficient (R) of all data is shown. **(c) **Simulated versus actual changes in occupancy of a TBP mutant that is defective in NC2 interactions. Production of HA-tagged TBP(F182V) in a wild-type TBP, *taf1*(*ΔTAND*) background was induced with galactose. After 45 minutes, cells were subjected to formaldehyde crosslinking. TBP was immunopurified and Cy3- or Cy5-labeled ChIP DNA was hybridized to yeast intergenic microarrays. The hybridization control reference used galactose-induced HA-tagged wild-type TBP in a *taf1*(*ΔTAND*) background, processed in parallel with TBP(F182V). Since the experimental set-up included both endogenous wild-type TBP and TBP(F182V) in the same cell, the simulation simultaneously ran the mechanism in duplicate in which the first mechanism was initialized with parameters for wild-type TBP and the second mechanism with parameters used to simulate the F182V mutation (Additional data file 3). The output being compared is the occupancy level of TBP(F182V) relative to TBP(WT). The line fit and correlation coefficient excludes the outlying cluster 5 data point.

To enhance the accuracy of the modeling and assist in identifying suitable values for the flux constants, we employed additional types of data. First, the flux constants were empirically set so as to recapitulate the relative transcription frequencies that have been experimentally measured [3] for genes in each of the six clusters. Next, we turned to measured promoter occupancy levels of individual components of the network using genome-wide ChIP-chip data [50]. We examined SAGA (Spt3 subunit), NC2 (Bur6 subunit), Mot1, TFIID (TAF1, TAF5, TAF6, and TAF9 subunits), TBP, and pol II (Rpb1, Rpb2, and Rpb7 subunits). For a particular cluster, the flux constants governing the 16 reaction segments shown in Figure 1 were adjusted such that a single set of values optimally recapitulated the relative occupancy level of each of these components as well as transcriptional output. Figure 4a compares simulated versus actual levels of RNA output and factor occupancy. The average correlation coefficient for these relationships was 0.8, which indicates a good fit between the model and the experimental data.

To simulate the experimental effect of each mutation that perturbs the TBP regulatory network, the flux constants for the relevant reaction segments were altered, and steady-state RNA output reported (see Additional data file 3). We allowed only those flux constants that are justifiably governed by the interaction being mutated to change, and only in the direction expected of the mutation, thereby constraining the degrees of freedom. For example, the impact of the NC2-defective TBP(F182V) mutation was simulated by reducing the flux constant for reaction segments 8 and 11, which govern the association of NC2 with TBP (illustrated in Figure 1). For multiple mutations, the relevant flux constants were set at or near to the values for the single mutations allowing an approximate three-fold tolerance. This tolerance attempts to accommodate the possibility that one mutation might influence the effect of other mutations.

Figure 4b compares simulated changes in RNA output upon network perturbation to experimentally measured changes when the network is perturbed by mutations. There was a remarkable correspondence (R = 0.8), indicating that the modeled changes can largely account for the experimentally observed changes. To assess the specificity of modeling we randomly assigned each set of cluster-specific values to a different cluster and re-ran the analysis. As expected, no substantial correlation was observed (data not shown), indicating that the flux parameters for each cluster are likely to generate a unique or limited number of solutions.

As a further test of the core mechanism illustrated in Figure 1, we examined whether a simulated perturbation to the TBP regulatory network could predict changes in TBP occupancy at promoters measured by ChIP-chip. Figure 4c compares the simulated and experimental data for changes in TBP occupancy in a *taf1*(*ΔTAND*) strain harboring both wild-type TBP and the TBP(F182V) mutation. With the exception of cluster 5, the simulation correlated with the experimental data (R = 0.56). The alternative outcome for cluster 5 illustrates the value of the simulation, in that it suggests that the mechanism and/or initialized flux constants need to be adjusted for cluster 5 genes, and thus requires further investigation. Deviation of the expression data from the simulated data for cluster 5 is also apparent in Figure 4b (red symbols). Taken together, the correlations between the simulated and experimental data for transcription frequency, ChIP-chip occupancy, and expression profiling suggest that the core mechanism illustrated in Figure 1 is a plausible prototype of the genome-wide TBP regulatory network.

### A TBP regulatory network tailored to sets of genes

**Figure 5:**
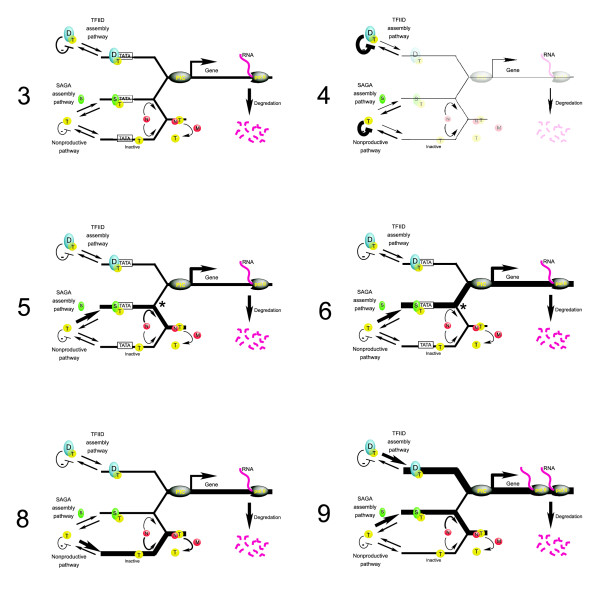
The 'flow' of PIC assembly through the TBP regulatory network at different gene clusters. Each assembly model corresponds to the indicated cluster number in Figure 3. The same prototype model described in Figure 1 is applied to all clusters. Line and arrow thickness reflect the magnitude of flux through the system.

Given the ability of the core mechanism to act as a template for simulating the behavior of each cluster, we next tailored the basic network model shown in Figure 1 to reflect actual behavior. In Figure 5, each model is drawn to reflect the 'flow' of TBP regulation that leads to PIC assembly and RNA production. Thicker arrows and thicker lines denote higher levels of flux through that portion of the network. We interpret these models as follows: PIC assembly at cluster 9 is dominated by the TFIID assembly pathway, and so the arrow governing TFIID association and the line representing the promoter are drawn thicker. The SAGA pathway also participates in cluster 9 but contributes little to RNA production because NC2 and Mot1 shunt this pathway in a nonproductive direction. Cluster 6 on the other hand is dominated by the SAGA pathway, possibly due to the presence of a TATA box at these genes (Table 1, row 17) that is linked to the SAGA pathway [23]. Cluster 8 is dominated by the nonproductive TBP pathway. Here, NC2 and Mot1 function positively in transcription by dismantling this inhibitory pathway. Cluster 4 genes are lowly expressed due in part to blockage of TBP by dimerization and TAF1 TAND interactions.

This TBP regulatory network accommodates much of the biochemical, genetic, and genomic data on TBP, making it particularly compelling and useful for rationalizing the functional significance of the clustering patterns and predicting experimental outcomes. In particular, the network has the following features. First, it allows the three assembly pathways (TFIID, SAGA, and nonproductive) illustrated in Figure 5 to operate in competition and, therefore, influence each other. Hence, regulation of the SAGA pathway can influence contributions by the TFIID pathway. Second, when one pathway is diminished, as in an *spt3Δ *strain, the other pathway is allowed to compensate, providing a limited degree of functional redundancy, as has been proposed for these two assembly pathways [21,22]. Third, inhibitors of TBP, such as Mot1 and NC2, can produce a net negative or net positive effect (or no effect) on transcription, as has been observed [25,26,33], depending upon which of the three assembly pathways predominately governs RNA output. Importantly, the model provides predictive power for designing, conceptualizing and analyzing genome-wide expression and ChIP microarray data as it relates to the TBP regulatory network.

### Design logic behind the TBP regulatory network

As demonstrated here, the flow of the TBP regulatory network is not uniform for all genes. Rather, it is tailored to specific genes. The question arises as to whether this tailoring is randomly placed or whether genes involved in a specific process utilize the TBP regulatory network in the same way. To address whether genes within a cluster have related functions, we examined if any of approximately 1,500 genome-wide properties that characterize yeast genes were particularly enriched within each cluster (Additional data file 4). A selected subset of these relationships is presented in Table 1. Most clusters were enriched with genes that belonged to a particular cellular process (rows 3-11). For example, cluster 4 was enriched with sporulation genes and cluster 8 with ribosomal protein (RP) genes. Thus, genes involved in the same process tend to be regulated by the TBP regulatory network in the same way. Below, we summarize some insights into the design logic.

**Figure 6:**
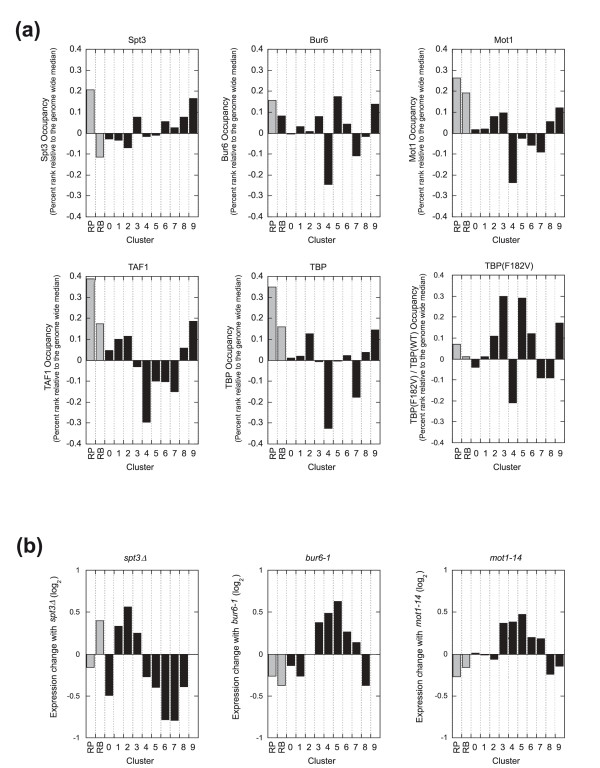
Properties of clusters 1-10 and RP and RB genes. **(a) **Bar graph comparison of promoter occupancy levels for Spt3, Bur6, Mot1, and Taf1 [50,52]. Values correspond to median percentile scores for each indicated gene group or cluster after subtracting the genome-wide median (defined as 0.5), and thus represent deviations from the median. RP and RB genes are explicitly defined in Table 1. **(b) **Bar graph plotting median changes in gene expression for each of the indicated gene groups or clusters in the presence of the indicated mutants.

RP genes encode physical components of the ribosome and are quintessentially TFIID-regulated and TATA-less [22,23,51]. However, computer modeling of RP-enriched cluster 8 genes (Figure 5, as well as the data in Figure 3) suggest that they are not only positively regulated by SAGA (Spt3) but are also positively regulated by the TBP inhibitors NC2 and Mot1. To assess the validity of these conclusions, we turned to published genome-wide ChIP-chip occupancy data of these factors and genome-wide expression profiles in NC2 and Mot1 mutants. Genome-wide promoter occupancy data of Spt3 (SAGA), Bur6 (NC2), Mot1, TAF1 (TFIID), and TBP [50,52] were transformed into percentile scores (ranging from 0 to 1) so that their relative contribution across clusters could be assessed. For each cluster from Figure 3 as well as for RP and ribosomal biogenesis (RB) genes, the bar graphs in Figure 6a display deviations from the genome-wide median (defined as 0.5). Strikingly, compared to all other clusters, the RP genes possessed the highest occupancy levels of SAGA, NC2, and Mot1 (as well as TFIID). However, RP genes displayed a rather modest transcriptional dependency on SAGA, NC2, and Mot1 (Figure 6b). Thus, the SAGA pathway assembles at RP genes but contributes modestly to transcription of these genes, which is consistent with the modeling of RP-enriched cluster 8 and 9 (Figure 5).

Clusters 3, 5, and 6 were enriched with genes involved in distinct metabolic processes, including amine metabolism, alcohol/sterol metabolism, and energy production, respectively. Genes in these clusters tended to rely on the more tightly controlled SAGA pathway and were negatively regulated by NC2 and Mot1 to varying degrees, resulting in distinct flow patterns in their assembly pathways (see Figure 5). The distinction between pathways 5 and 6 might indicate the presence of a regulatory step between TBP loading and PIC assembly (see asterisks in Figure 5), which is in line with previous suggestions of post-recruitment regulation [53,54].

Cluster 4 genes are very lowly expressed during exponential growth as might be expected of their enrichment with mid-phase sporulation genes (Table 1, rows 9 and 12). These genes appear to be inhibited in part by the functionally redundant action of TBP dimerization and the TAF1 TAND domain, and thus are related to cluster 4 in [42]. They tend to reside in the repressive environment of subtelomeric regions, where they are generally inhibited by the Hda1 histone deacetylase, the Ssn-Tup1 complex, and the methyltransferase Set1 (Table 1, rows 31-33). Cluster 4 genes, therefore, are likely to be maintained in a low transcriptional state by a variety of repression mechanisms, including those in the TBP regulatory network. The multiple levels of repression associated with cluster 4 genes might reflect the critical need to keep potentially detrimental sporulation genes inactive during vegetative growth. Consistent with their potential toxicity, any combination of mutants used in this study that resulted in elevated expression of cluster 4 was also found to be toxic to cell growth (Figure S3 in Additional data file 1).

The boundaries of SIR-mediated (Silent Information Regulator) repression extending from telomeric into subtelomeric regions along the chromosome are thought to be defined in part by the counteraction of bromodomain factor Bdf1 [55]. Since cluster 4 genes typically reside in these regions [42], we were surprised to find that they were not particularly repressed by SIR proteins or activated by Bdf1. They were, however, highly dependent upon *BDF2 *(Table 1, row 24), which is the paralog of *BDF1*. Little is known about *BDF2*, except that it is nonessential when *BDF1 *is present, and loss of this gene affects the expression of very few genes [55-57]. The linkage between cluster 4 and genes that tend to be more Bdf2-dependent provides a novel distinguishing feature between Bdf1 and Bdf2. While Bdf1 might be specialized to counteract SIR-mediated repression [55], in addition to its function with TFIID, Bdf2 might be particularly suited to operate in repressive environments that do not involve SIR repression.

## Conclusion

The study presented here suggests how the TBP regulatory network might be constructed. Rather than TBP regulators functioning equivalently at all genes or selectively at nonoverlapping sets of genes, they take on net negative or positive roles depending upon many factors, including whether TFIID or SAGA regulation predominates, and where the rate-determining step lies. Importantly, a single TBP regulatory network is applicable genome-wide, with genes involved in related biological processes being regulated by specific nodes in the network. Gene-specific regulators are likely to control the 'flow' of assembly through specific nodes. This TBP regulatory network is necessarily part of a much larger global gene regulatory network that includes regulation of activators, chromatin, and transcription elongation.

## Materials and methods

### Strains and plasmids

All strains were derived from Y13.2 (*MATα ura3-52 trp1Δ-63 leu2*, *3-112 his3-609 taf145Δ pYN1-TAF1*) [58]. The wild-type and ΔTAND strains have been previously described [42]. The *spt3Δ *and ΔTAND *spt3Δ *strains were constructed as derivatives of the above strains by replacement of the endogenous *SPT3 *gene with a PCR amplified *kanMX *cassette through homologous recombination [59]. Single mutant plasmids carrying the galactose inducible version of TBP on a CEN/ARS plasmid have been previously described or were constructed by site-directed mutagenesis from the wild type [47]. Double and triple mutant combinations were constructed by standard sub-cloning methods from the single mutant plasmids or by using site-directed mutagenesis when there were no suitable restriction enzyme sites available. The 2u version of the wild-type TBP plasmid was constructed through sub-cloning by exchanging a *Sca*I/*Cla*I fragment containing the CEN/ARS replication origin with a *Sca*I/*Cla*I fragment containing the 2u origin found in the pRS425 plasmid. This created p2uLF-yTBP(wt)(GAL10), which the various TBP mutants were introduced into by sub-cloning. All newly created plasmids were verified by sequencing.

### Cell growth for microarrays and immunoblot analysis

Transformants carrying the TBP plasmids were grown in CSM-Leu-Trp + 3% raffinose liquid media until they reached an OD_600 _= 0.65-0.8. Aliquots were removed (-galactose) for immunoblot analysis (Figure S2 in Additional data file 1). Expression of the TBP was induced by addition of galactose to a final concentration of 2%. The wild-type and ΔTAND strains contained the CEN/ARS version of the plasmids, which was induced for 45 minutes while the *spt3Δ *and ΔTAND *spt3Δ *strains carried the 2u version of the plasmids, which was induced for three hours. After induction, an aliquot (+ galactose) was removed prior to harvest. Anti-yTBP immunoblotting was performed on the -/+ galactose aliquots to monitor expression of the endogenous and Gal-inducible TBPs.

### Microarray expression analysis

Genome-wide changes in mRNA levels were measured using microarrays containing spotted PCR products of every open reading frame (ORF). The reference sample for all arrays was a wild-type strain expressing a null TBP that was treated identically and in parallel with the test samples. Cell harvesting, RNA extraction, and hybridization was performed as described previously [42]. Expression analyses of all test versus reference combinations were repeated at least twice, incorporating a dye-swap of the test and reference samples, with mRNA isolated from two independent transformants (biological replicates). Data were mode normalized and filtered for significant changes in gene expression essentially as described [42]. First, the signal intensities for each channel, as determined by subtracting the background median from the foreground mean signal, was required to be greater than 1 standard deviation above the local background signal. Second, ratios changed in the same direction in each replicate. Third, the average log ratio of replicates was at least two standard deviations from the mean ratio for that gene in the homotypic data set. The value used for the standard deviation was the greater of either the gene specific standard deviation or the pooled (all genes) standard deviation. Fourth, *p *values of the average log ratio when compared to the homotypic data set were <0.005. These criteria resulted in very few false positives when applied to independent homotypic experiments. Figure 3 included additional filters: fifth, fold changes in gene expression were >1.5; and sixth, data were present in 60% of the 63 experiments for any gene. Processed data are available in Additional data file 2. A portion of the data from Figure 3 was obtained from [42,48]. Raw data are accessible at GEO [60] under series accession number GSE7385. Expression data for *bur6-1 *and *mot1-14 *was obtained from [25,30].

### Genome-wide ChIP-chip analysis

Cell growth for TBP(F182V) ChIP-chip analysis was identical to cell growth for the expression analysis. After the 45 minute galactose induction, cultures were immediately crosslinked at 25°C for 2 h with 1% formaldehyde. The test strain contained TBP(F182V) and the reference strain contained wild-type TBP, both in a *taf1*(*ΔTAND*) strain harboring endogenous wild-type TBP. Two biological replicates were hybridized to microarrays containing spotted PCR amplified intergenic regions, as described [23], with the following modifications. Sonication was performed for 18 sessions and after removal of an input sample. Galactose-induced TBP was immunoprecipitated with hemagglutinin (HA) antibodies. Signal intensities were calculated as described for expression arrays and filtered to remove any spots whose signal in either channel was less than one standard deviation above local background. The data for promoter-containing intergenic regions was normalized by setting the log_2 _ratio of all nonpromoter containing intergenic regions (tail-to-tail regions) equal to zero, followed by averaging the normalized log_2 _ratios of replicates.

Genome-wide occupancy data for SAGA (Spt3), NC2 (Bur6), Mot1, TFIID (TAF1, TAF5, TAF6, TAF9), and pol II (Rpb1, Rpb2, Rpb7) were obtained from [50,52]. Occupancy values are defined as the ratio of the chIP signal/control signal, where the control represents signal generated from nonspecific contamination of genomic DNA during immunoprecipitation using the method described in reference [61].

### Relationship analysis

The relationships to the top and bottom 10% of the expression and ChIP-chip distributions (Additional data file 4) were calculated in Excel with the data downloaded from the referenced lab or journal's websites. The percent rank of the distribution was calculated with the PERCENTRANK function. Then the number of genes that appear in the top 10% (>0.9 in PERCENTRANK) or the bottom 10% (<0.1 in PERCENTRANK) and appear in each cluster were calculated. The CHITEST function of Excel was then used to calculate *p *values from the observed and expected values. *P *values for rows 5-11 in Table 1 were calculated by GO term finder at the *Saccharomyces *Genome Database [62].

## Additional data files

The following additional data are available with the online version of this paper. Additional data file 1 is a PDF containing supporting text and figures. Additional data file 2 is an Excel workbook that contains the log_2 _ratios of fold changes in gene expression for the data shown in Figure 3 (63 × 6,227 expression ratios), the standard deviation of each cluster, and ChIP-chip log_2 _ratios of occupancy relative to wild-type TBP for experiments that were not published elsewhere (that is, Figure 4c). Additional data file 3 is an Excel workbook containing the flux constant values for each cluster and the products of the simulation. Additional data file 4 is an Excel workbook containing *p *values for overlapping relationships between gene clusters described here and a large amount of published genomic data.

## Supplementary Material

Additional data file 1 Supporting text and figures. Click here for file

Additional data file 2 Log_2 _ratios of fold changes in gene expression for the data shown in Figure 3 (63 × 6,227 expression ratios), the standard deviation of each cluster, and ChIP-chip log_2 _ratios of occupancy relative to wild-type TBP for experiments that were not published elsewhere (that is, Figure 4c). Click here for file

Additional data file 3 Flux constant values for each cluster and the products of the simulation. Click here for file

Additional data file 4 *P *values for overlapping relationships between gene clusters described here and a large amount of published genomic data. Click here for file

## Figures and Tables

**Table 1:** Significance values relating genomic properties to clusters defined by the TBP regulatory network*

		Cluster number^†^									
		
Row^‡^	Property	0	1	2	3	4	5	6	7	8	9
	**Environmental stress**										
1	Induced during heat stress	-25	-6	...	8	...	30	35	...	...	...
2	Repressed during heat stress	-7	106	...	...	...	...	...	...	...	...
											
	**Gene ontology**^§^										
3	RB genes^¶^	...	69	...	...	...	...	...	...	...	...
4	RP genes^¶^	...	...	...	...	-4	...	...	...	31	14
5	Oxidative phosphorylation {GO:6119}	...	...	...	...	...	...	...	...	...	13
6	Amine metabolism {GO:9308}	...	...	...	8	...	...	...	...	...	...
7	Alcohol metabolism {GO:6066}	...	...	...	5	...	9	4	...	...	...
8	Energy derivation {GO:15980}	...	...	...	...	...	6	13	...	...	5
9	Sporulation {GO:30435}	...	...	...	...	4	...	...	...	...	...
10	Ty element transposition {GO:6319}	...	...	...	...	...	...	...	6	...	...
11	Proteolysis {GO:6508}	...	...	6	...	...	...	...	...	...	...
											
	**General properties**										
12	Lowly transcribed	84	...	...	...	89	...	...	10	...	...
13	Highly transcribed	...	...	...	...	-7	...	...	...	12	21
14	Low mRNA levels	14	...	...	...	28	-5	-6	...	...	...
15	High mRNA levels	-7	-6	...	...	-7	...	32	...	8	21
16	SAGA-dominated	-46	...	...	9	...	53	43	...	...	...
17	TATA-containing	-52	-6	...	9	12	45	46	...	...	4
18	Low H3 K4Me2 in ORF	...	...	...	...	58	...	...	...	...	...
19	Low H3 K4Me2 in IGR	...	...	...	...	28	...	...	...	...	...
											
	**Positively regulated by**										
20	Spt3 (SAGA)	-31	-4	-7	...	28	5	31	28	...	...
21	Mot1	...	...	...	...	-4	-6	...	...	27	8
22	Bur6 (NC2)	13	15	...	...	...	-5	...	...	17	...
23	Bdf1 (TFIID)	11	...	...	...	...	...	...	10	6	...
24	Bdf2	...	...	...	...	20	...	...	...	...	...
25	TAF1 (TFIID)	...	...	...	...	...	...	...	...	10	5
											
	**Negatively regulated by**										
26	RP genes	...	^¥^	...	...	...	...	...	...	...	...
27	Spt3 (SAGA)	-8	6	27	30	...	...	...	-5	...	...
28	Mot1	-25	...	...	15	22	36	5	...	-6	...
29	Bur6 (NC2)	-28	...	...	5	13	43	9	...	-4	...
30	Bdf1 (TFIID)	-21	...	...	10	...	25	8	...	...	...
31	Hda1	-23	...	...	...	25	11	5	5	...	...
32	Tup1 (SSN6-TUP1)	-31	...	-4	...	52	12	11	17	...	...
33	Set1 (COMPASS)	-8	...	-6	...	60	...	...	15	...	...

*Values represent -log_10 _*p *values, using the extreme most 10% of the data points (n > approximately 3,000) as identified in Additional data file 4, and are calculated using the CHITEST formula in EXCEL. Negative signs are inserted where relationships are anti-correlated. For clarity, any absolute value less than 4 was replaced with a period. ^†^Cluster numbers refer to clusters shown in Figure 3. Cluster 0 includes all genes not clustered in Figure 3. ^‡^Row identifier in the text. References are provided in Additional data file 4. ^§^Gene Ontology term identifier is provided in brackets. *P *values were derived using 'GO term finder' [62]. ^¶^RP genes are defined here as genes defined by the following Gene Ontology terms: ribosomal protein, mitochondrial ribosomal protein, translational elongation, translational initiation, translational termination, protein biosynthesis. RB genes are defined here by the following Gene Ontology terms: 35S primary transcript processing, ribosomal large subunit assembly and maintenance, rRNA metabolism, rRNA modification, rRNA processing, rRNA nucleus export, transcription from Pol I promoter, tRNA methylation, tRNA modification, tRNA processing, tRNA splicing, tRNA nucleus export, regulation of translation, processing of 20S pre-rRNA. ^¥^Up-regulated upon deletion of *RPL12A *(*p *< 10^-24^), *RPL27A *(*p *< 10^-26^), *RPL34A *(*p *< 10^-8^), *RPL6B *(*p *< 10^-9^), *RPL8A *(*p *< 10^-63^), or *RPL24A *(*p *< 10^-20^); see entries R135T, R137T, R138T, R139T, R140T, R141T in Additional data file 4.
