# Supplementary material for: A TATA binding protein regulatory network that governs transcription complex assembly
Source: Genome Biol. 2007 Apr 2;8(4):R46. doi: 10.1186/gb-2007-8-4-r46 (PMC1896006; doi:10.1186/gb-2007-8-4-r46)
Supplement: Additional data file 1 — Supporting text and figures. [file gb-2007-8-4-r46-S1.pdf]

Additional data file 1

Huisinga and Pugh, Genome Biology (2007)

See main text for references

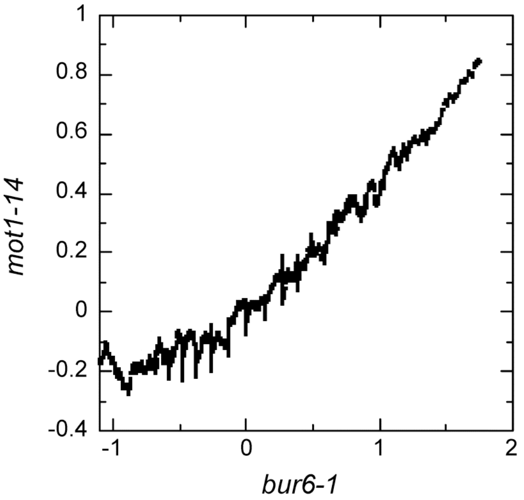

Figure S1. Sliding window correlation plots of genome-wide changes in gene expression between *bur6-1* and *mot1-14* mutants. Data were averaged over 100-gene sliding windows taken in 1 step increments.

Computational simulation definitions

Definition of reaction mechanism terms: *(in order of appearance)*

IFT	TFIID complex, containing TBP, and inhibited by the TAF1 TAND domain
FT	Monomeric TFIID complex
FTFT	Self-associated TFIID dimer (presumably via the TBP subunit), which is incapable of binding DNA
TT	Self-associated TBP dimer, which is incapable of binding DNA
T	Monomeric TBP
P	Promoter DNA
FTP	TFIID bound to promoter DNA
S	SAGA complex, containing Spt3
ST	SAGA complex bound to TBP
STP	SAGA•TBP complex bound to promoter DNA
TP	TBP nonproductively bound to promoter DNA
N	NC2 complex, containing Bur6
NTP	NC2•TBP complex bound to promoter DNA
M	Mot1
MNTP	Mot1•NC2•TBP complex bound to promoter DNA
PIC1	Pre-initiation complex containing TFIID and pol II (and other proteins)
PIC2	Pre-initiation complex containing SAGA and pol II (and other proteins)
RNA	mRNA product derived from PIC firing
X	Degraded RNA

All values for flux constants, initial concentrations, and outputs are reported in additional data file 3.

All reactions were run to steady-state (500 sec.)

Supporting Results

Section 1: Experimental perturbation of the TBP regulatory network

The complete interaction interface between TBP and its regulators has not been fully defined, despite TBP being a well-mapped components of the transcription machinery. Ideally, we would prefer to eliminate interactions by mutating a single amino acid on the relevant interaction surface of TBP. This was not feasible for Spt3 or the TAF1 TAND domain. Since Spt3's physical interaction surface on TBP, if any, has not been defined, it was necessary to delete the entire nonessential *SPT3* gene. Deletion of TBP regulatory subunits *SPT7* or *SPT20* gave essentially the same changes in gene expression as deletion of *SPT3*, suggesting that the effects of *SPT3* are due to loss of TBP-directed SAGA functions (data not shown). A TAND interaction surface on TBP has been defined [16, 17, 58], but it overlaps with TBP's DNA binding and dimerization interface. TBP mutations that affect TAND interactions do not elicit the same phenotype as a TAND deletion, and so a TAND deletion was used to ensure that TAND function was eliminated. TBP mutations F182V, K145E, E93R (or R107E, N2-1), V161E, and V161R were used to impair interactions with NC2, Mot1, TFIIA, DNA, and TBP homodimers, respectively (Figure 2c, main text). Since these mutations generally do not support cell viability it was necessary to place them in strains that also harbored wild type TBP. These mutants were therefore required to compete with the endogenous TBP. Their general ability to do so, and evidence that these mutations primarily impair the indicated interactions is provided in references [46, 47, 64-66] and below.

The TBP mutants were placed under control of the *GAL10* promoter (illustrated in Figure 2c, main text), and were inducible upon addition of galactose (Figure S2). This provided the advantage of examining genome-wide impacts on expression in normal cells soon after "poisoning" the TBP regulatory network, rather than after prolonged periods where potential physiological compensation by the cell could obscure direct effects from indirect effects. We chose not to use a shut-off approach involving temperature-sensitive alleles since not all regulatory interactions under study could be selectively eliminated by such alleles, and heat shock itself causes genome-wide re-programming that differentially impacts the SAGA and TFIID assembly pathways illustrated in Figure 1 of the main text [22].

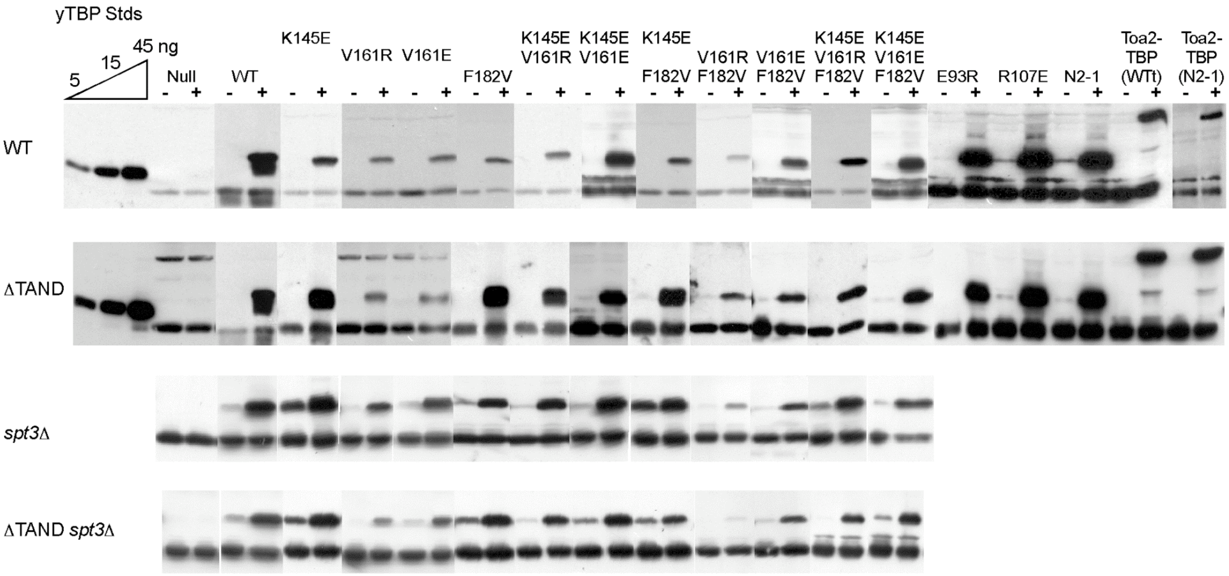

Figure S2. Detection of galactose-induced TBP. Cultures were grown in raffinose and induced for 45 min (or 3 hrs in *spt3Δ* strains) with 2% galactose. "- +" indicate before and after galactose induction, respectively. Blots containing 0.5 OD_{600} of solubilized whole cells were probed with TBP antibodies. Recombinant his-tagged TBP quantitation standards are shown to the left. In each row of images, the endogenous TBP electrophoreses towards the bottom of the gel, whereas the TBP mutants run slower due to the presence of the HA tag. HA-tagged TBP levels should be normalized to endogenous TBP levels to obtain more accurate estimates. Endogenous TBP levels generally remain constant in all mutants. Apparent differences in endogenous TBP levels reflect differences in film exposures or detection efficiency from the ECL.

In an effort to determine whether the experimental strategy was likely be fruitful in perturbing global gene expression patterns, we first assessed whether any mutant combination dominantly impacted cell growth. Strains containing or lacking the TAF1 TAND domain and/or *SPT3* were serially diluted and plated onto galactose-containing media (Figure S3). Several combinations of TBP mutations impaired cell growth, suggesting that they are likely to impact gene expression patterns.

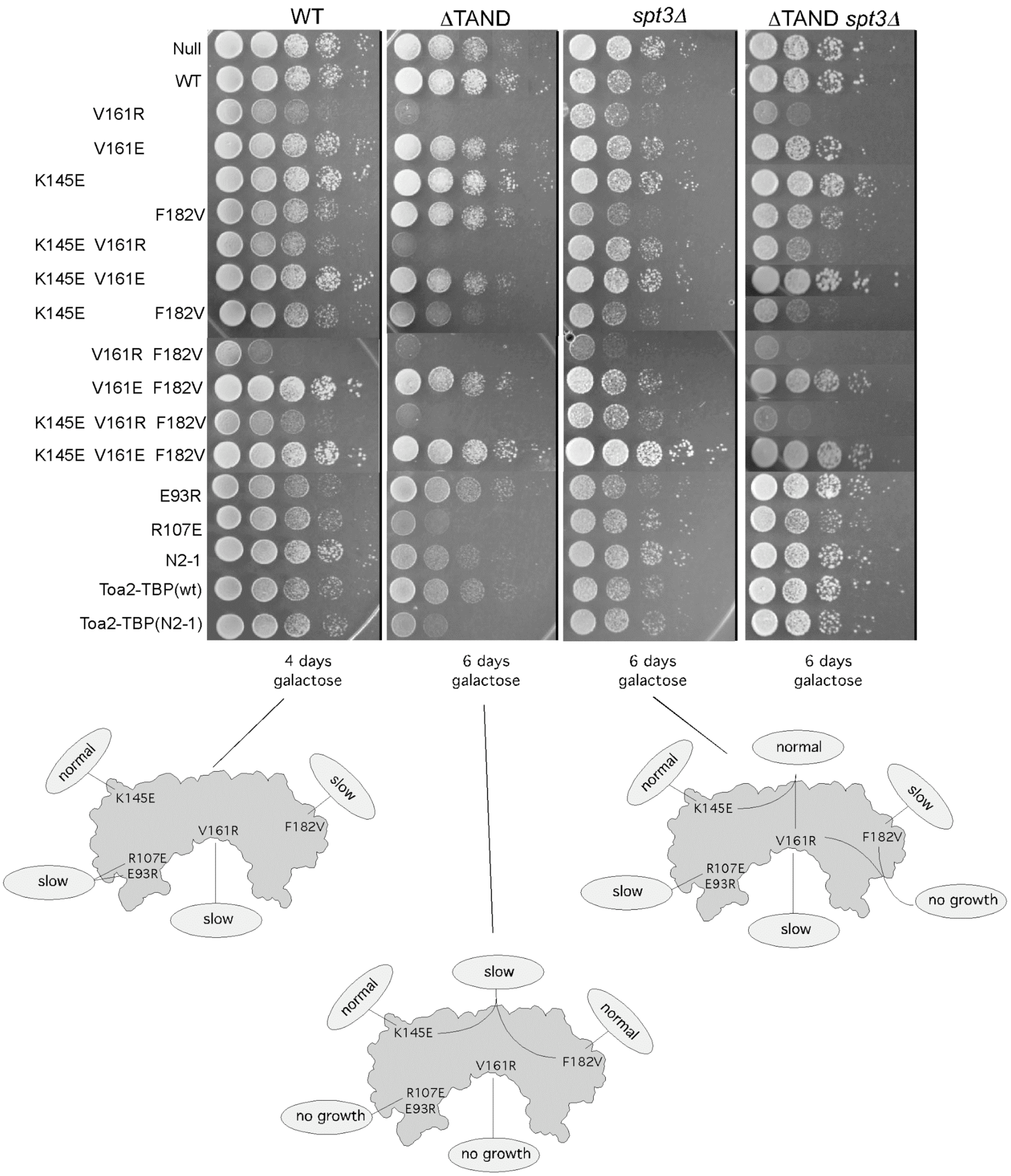

Figure S3. Growth of yeast strains upon disruption of parts of the TBP regulatory network. Yeast strains having the genotype indicated above each panel were transformed with a TBP-expressing plasmid bearing the mutations indicated on the left side. Strains were serially diluted and spotted from left to right onto solid media containing galactose. Plates were incubated at 30˚C for the number of days indicated below each panel. Notable dominant growth phenotypes in wild type, *taf1(ΔTAND)* and *spt3Δ* strains are mapped onto TBP's structure. Note, all strains contain endogenous wild type TBP.

Figure S3 method: CEN/ARS plasmids expressing various TBP derivatives under control of the *GAL10* promoter were transformed into wild-type, *Δ*TAND, *spt3Δ* and *Δ*TAND *spt3Δ* strains. Transformants were selected on CSM-Leu-Trp + 2% glucose, and subsequently grown in CSM-Leu-Trp + 3% raffinose liquid media. At OD_{600} = 1.0, ten microliters of washed cells, or serial 10-fold dilutions, were plated onto CSM-Leu-Trp + 2% galactose or glucose agar (glucose data not shown).

TBP mutants that are defective for dimerization/DNA binding (V161R) or for interactions with NC2 (F182V) or TFIIA (E93R or R107E) resulted in dominant slow growth phenotypes, reflecting the importance of these interactions in maintaining proper gene expression. The NC2-interaction mutant (F182V) was toxic when combined with a Mot1-interaction mutant (K145E) in a *taf1(ΔTAND)* strain. Thus, regulation of gene expression by the TAF1 TAND domain, along the TFIID assembly pathway, is likely to be highly intertwined and in some cases redundant with other TBP regulators including some such as NC2 and Mot1 that are associated with the distinctly different SAGA transcription regulatory pathway.

When SAGA's regulatory interaction with TBP was removed using the *spt3Δ* strain, many of the TBP mutants behaved as they did in the wild type *SPT3* strain, with two exceptions. The V161R, F182V double combination was severely toxic, and K145E suppressed the toxicity associated with the V161R mutation, including the V161R F182V double mutant. These types of genetic interactions suggest a complex interplay of TBP regulators, many of which are only revealed after disruption of multiple interactions. Interestingly, expression of certain TBP mutants in the *taf1(ΔTAND) spt3Δ* double mutant lead to heterogeneous cell growth (colonies of various sizes) and chromosomal instability (not shown), suggesting that loss of a number of TBP regulatory interactions leads to a breakdown of the network.

Section 2: Validation of the TBP mutants

As a first step towards interpreting the microarray data presented in the main text, we sought to re-affirm that each TBP mutation was likely to be affecting the targeted interaction. Previously, we provided evidence that V161E predominately affected DNA binding, and V161R affected both DNA binding and dimerization in vitro and in vivo [47]. By comparing the two, the contribution from dimerization could be deduced.

Defects in NC2 interactions. To ascertain whether the F182V mutation, in our experimental context, was consistent with a loss of NC2 interactions, several comparisons were made. First, we compared the changes in expression in the TBP(F182V) mutant with that of a *bur6-1* temperature-sensitive NC2 mutant from Cang et al. [25]. Bur6 is a subunit of NC2. As shown in Figure S4a, subpanel 1, the changes were well-correlated despite the two experiments being conducted under different conditions. For a negative control comparison, TBP(F182V) correlated poorly with *taf5-9* (Figure S4a subpanel 2), a temperature-sensitive TFIID mutant, which is not expected to have a relationship with TBP(F182V). For a positive control comparison, TBP(F182V) was compared to itself in subpanel 3, but in two different strains, WT and *taf1(ΔTAND)*.

Second, we assessed whether the constitutive presence of endogenous wild type TBP in our study affected the genome-wide expression pattern in the TBP(F182V) mutant. TBP(F182V) supports cell viability in the absence of wild type TBP and is temperature sensitive. We compared genome-wide expression profiles for TBP(F182V) in the presence (our data) and absence (Cang et al. data) of endogenous TBP. Again a good correlation was obtained despite the experiments being performed in different laboratories (Figure S4a subpanel 4).

The third comparison evaluated whether changes in gene expression caused by the F182V mutation were directly linked to changes in promoter binding by this mutant. In the absence of NC2 interactions, TBP(F182V) is expected to display increased promoter occupancy

and a concomitant increase in transcription. We performed genome-wide chromatin immunoprecipitation (ChIP-chip) assays, whereby changes in occupancy of the TBP(F182V) mutant in a *taf1(ΔTAND)* strain was measured at ~6000 promoter regions of the yeast genome relative to the binding of wild type TBP in a wild type strain. The *taf1(ΔTAND)* strain was used because it displayed the highest sensitivity to the F182V mutation of the strains tested. Changes in occupancy of TBP(F182V) were compared with changes in transcriptional output from the same strain (Figure S4b, subpanel 1). Both were well-correlated compared to a strain lacking TBP(F182V) (subpanel 2). Thus, changes in transcriptional output caused by the F182V mutation in general appear to be a consequence of this mutant binding directly to target promoters.

Defects in Mot1 interactions. The K145E mutation impairs TBP interactions with Mot1 and TFIIA, in vitro [46, 64]. The TBP(K145E) mutant showed little change in gene expression unless placed in the context of other mutations such as *taf1(ΔTAND).* To assess whether TBP(K145E) was primarily defective in Mot1 or TFIIA interactions in vivo, two comparisons were made. First, we compared the genome-wide expression profile of TBP(K145E) in a *taf1(ΔTAND)* strain to a *mot1-14* mutant for those genes residing in clusters where K145E was having an impact. As shown in Figure S4c subpanel 1, changes in gene expression in a strain in which TBP(K145E) was induced with galactose correlated well to changes occurring in a *mot1-14* strain. In comparison, changes in expression in the *mot1-14* strain did not correlate with a galactose-induced TFIIA-defective TBP(R93E) mutant (subpanel 2).

In the second comparison, relevant hierarchical relationships from Figure 3 of the main text were redrawn in Figure S4d, where TBP mutants that are expected to have altered interactions with TFIIA or NC2 were compared to the K145E mutation. These include E93R and R107E which lie at the crystallographic TFIIA-TBP interface [67, 68], the N2-1 mutant which has been previously described as defective in TFIIA interactions [66], and Toa2-TBP which is a fusion of the Toa2 subunit of TFIIA to the amino-terminal end of TBP [66]. These mutants clustered together away from K145E. In fact, K145E clustered closer to F182V which is consistent with the known functional linkage between Mot1 and NC2. Taken together, these data affirm that the K145E mutation alters the functional interactions between TBP and Mot1 more than with TFIIA in vivo.

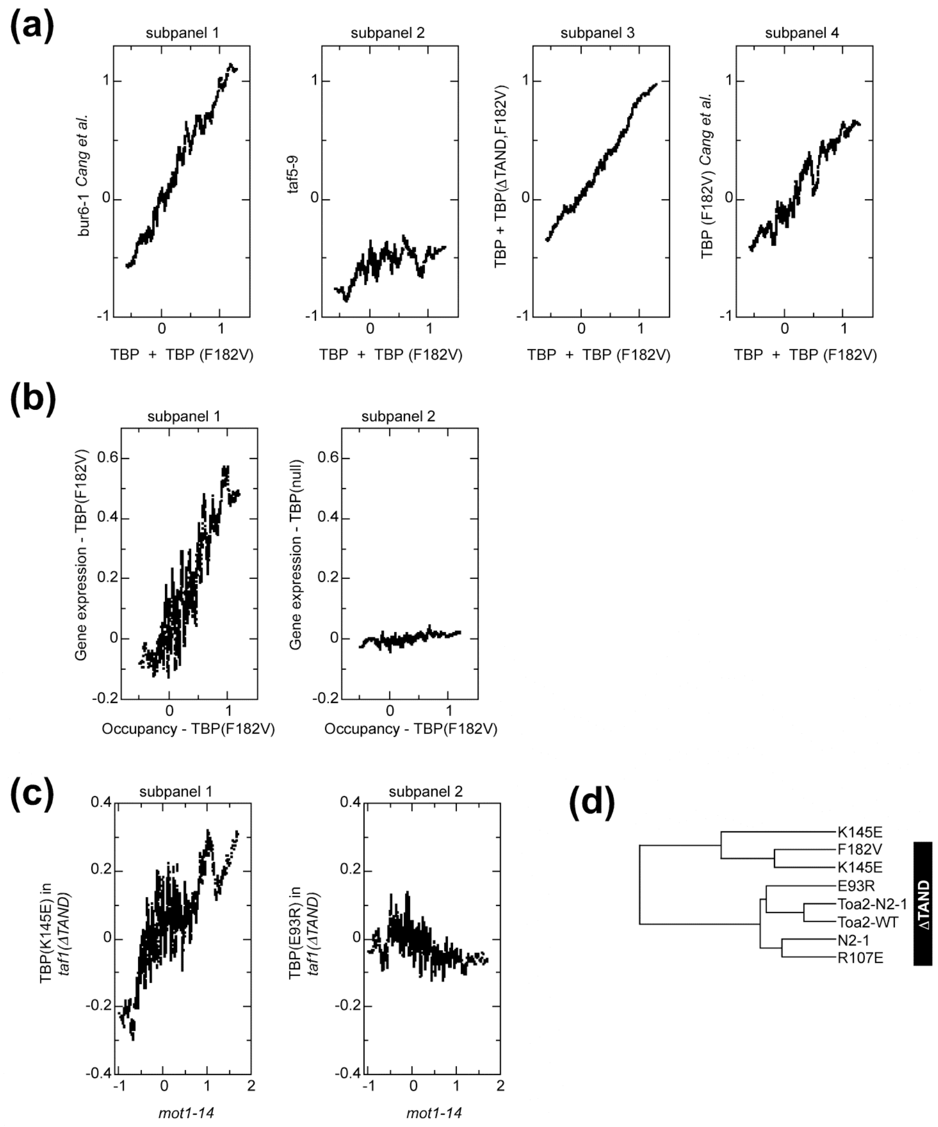

Figure S4. Validation of the TBP mutants.

(a) Sliding window correlation plots of genome-wide changes in gene expression of the TBP(F182V) mutant vs. published NC2-TBP interaction defective mutants. Data sets were derived from Figure 3, and published data sets were obtained from [25]. All axes represent $\log_2$ changes in gene expression averaged over 100-gene sliding windows taken in 1 step increments.

(b) Sliding window correlation plots of genome-wide changes in promoter occupancy of the TBP(F182V) mutant vs. corresponding gene expression changes. Chromatin immunoprecipitation or expression assays were performed on a genome-wide scale for TBP(F182V) (*left panel*) or TBP(null) (*right panel*) in a *taf1(ΔTAND)* strain. Log_2 changes in occupancy or expression are relative to TBP(WT) in a TAF1(WT) strain, and were filtered to include only those genes present in Figure 3. Data were averaged over 100-gene sliding windows taken in 1 step increments.

(c) Sliding window correlation plots of genome-wide changes in gene expression between TBP(K145E) (*left panel*) or TBP(R93E) (*right panel*) and *mot1-14*. Log_2 changes in gene expression were derived from Figure 3, clusters 3, 5, 8, and 9. The *mot1-14* data set was from reference [30]. Data were averaged over 100-gene sliding windows taken in 1 step increments.

(d) Dendogram of TBP mutants. Dendogram branches for the indicated mutants were taken from Figure 3 of the main text.

Section 3: Clusters arising from potential indirect effects

Potential indirect effects associated with cluster 1. The genes in cluster 1 had the unique property of being up-regulated in response to deletion of RP (ribosomal protein) genes, including *RPL8A*, *RPL27A*, *RPL12A*, *RPS24A*, and *RPL6B* (Table 1, main text). Ribosomal proteins are not known to directly regulate gene expression. Conceivably, lower ribosome levels due to deletion of RP genes or lower RP mRNA might indirectly up-regulate RB (ribosomal biogenesis) genes. If the cell senses that it is not making enough ribosomes to meet cellular needs, then it is logical for the cell to increase production of proteins involved in ribosomal biogenesis. If true, then perturbations that decrease RP gene transcription might create the secondary effect of increasing expression of cluster 1 genes. Consistent with this possibility, many of the interactions that positively regulated the RP genes in clusters 8, negatively regulated cluster 1 genes, resulting in an approximate green/red mirror image of the cluster pattern (see Figure 3 of the main text), and an inverse correlation (Figure S5). One exception was the TBP F182V mutation which caused a drop in cluster 8 expression but not a corresponding increase in cluster 1 expression. However, unlike the other TBP regulators, NC2 (Bur6) positively regulated cluster 1 genes (Table 1, row 22). We speculate that loss of NC2 function at cluster 8 has the potential to give rise to increased expression of cluster 1, but that this did not materialize without a positive contribution of NC2 at cluster 1 genes.

Taken together, the data suggest that cluster 1 genes, which include RB genes, are not directly inhibited by the SAGA pathway but instead are sensing and responding inversely to RP gene expression (including mRNA and/or protein levels). The rationale for this type of regulation might lie in distinct cellular roles for RP and RB genes. RP gene products are abundant structural proteins, whereas many RB gene products might act catalytically and thus are required at lower levels. Consequently, the genes in clusters 8 and 9 are hyper-transcribed, which is a task the SAGA pathway is well suited for.

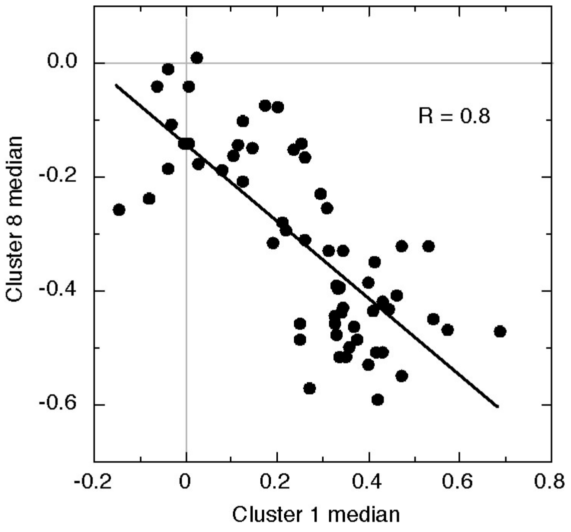

Figure S5. Changes in gene expression for cluster 8 is inversely correlated with changes in cluster 1. The median $\log_2$ change in gene expression for clusters 1 and 8 were determined for each of the 63 sets of microarray experiments, and plotted against each other. A linear fit to the data produces a correlation coefficient of 0.8.

Potential indirect effects associated with cluster 10. The genes in cluster 10 are almost entirely found throughout chromosomes XI and XII (Figure S6), suggesting that these chromosomes underwent duplication in these strains. These apparent duplications arose spontaneously in individual colonies or during culturing in liquid media after transformation of the parent strain with the TBP mutants, and thus were not stably propagated and were not assayed for gene copy number. Although included in the clustering, these mutants had little effect on the distribution of genes in clusters 1-9.

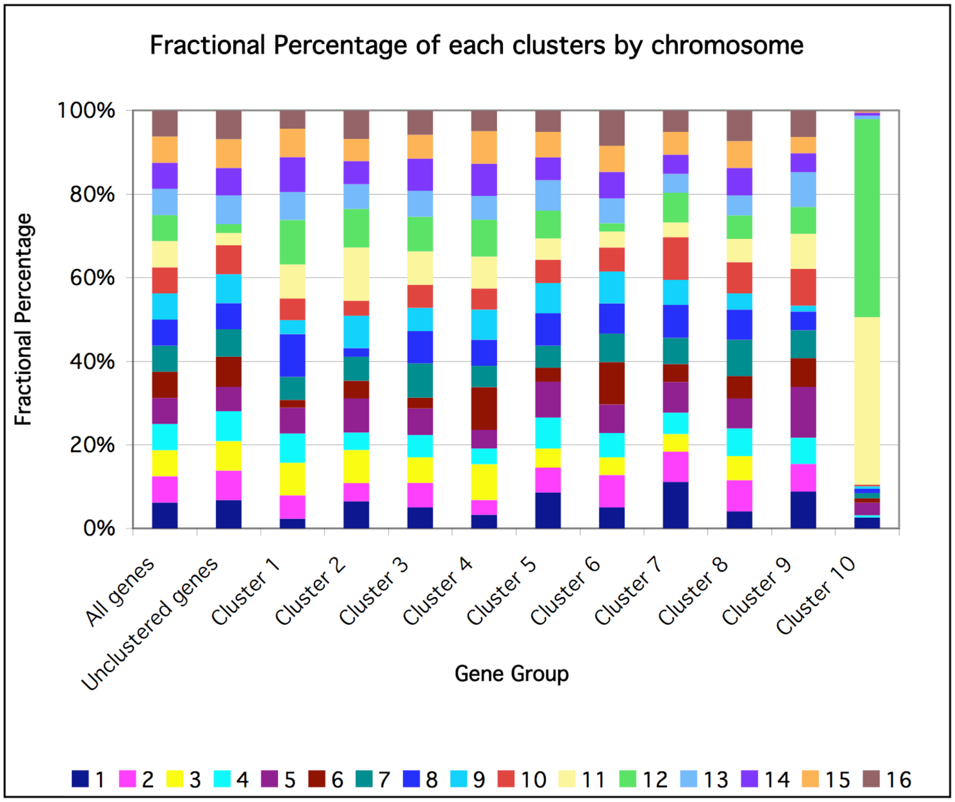

Figure S6. Genes in Cluster 10 are over-represented on chromosomes XI and XII. Each chromosome (numbered 1 through 16) is indicated by a different color in the stacked bar graph. Percentage of genes in clusters from each chromosome. Each cluster should be compared to the genome-wide distribution (All genes). Cluster 10 is extremely over-represented for genes from chromosomes XI and XII while the other clusters are generally unbiased.

Perturbing the TBP regulatory network does not cause a general stress response. Several clusters were enriched with genes that are either induced or repressed by heat stress (Table 1, rows 1 and 2, main text). Therefore the question arises as to whether simply expressing defective TBP mutants or constitutively mutant forms of SAGA and/or TFIID elicits a stress response, which could in principle account for the observed changes in gene expression. Several observations indicate that a general stress response is not the primary cause of changes in gene expression in these mutants, although some level of influence cannot be excluded. First, as Figure 3 in the main text demonstrates, not all mutations lead to similar changes in gene expression, as would be expected if each were eliciting the same stress response. Instead, many mutants affect distinct sets of genes. Second, none of the mutants mimic a typical stress response profile, as shown in Figure S7, where the median expression changes for three potentially relevant stress responses (an unfolded protein response caused by tunicamycin, overexpression of H2Kbeta, and a 20 min heat shock) is compared with two very distinct TBP mutant profiles (V161R in *taf1(ΔTAND)* and F182V). In many cases, the TBP mutants elicited expression changes that were opposite to that of a stress response. Third, stress responses are typically transient and thus are unlikely to be maintained in strains (e.g. *spt3Δ*) harboring constitutive deletions.

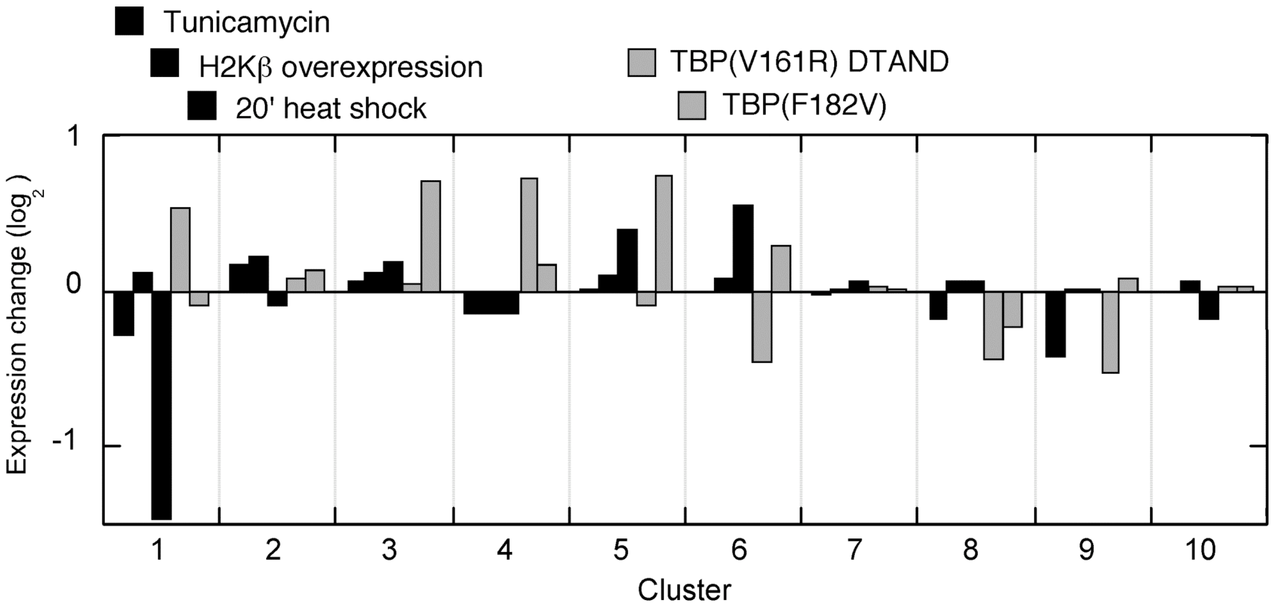

Figure S7. Three stress responses are compared to two distinct responses from TBP mutants that are galactose-induced. Log_2 fold changes in gene expression are reported. The three stress data sets are Tunicamycin [69], H2Kb overexpression [69], and a 20 min heat shock [70]. The TBP data sets are TBP(V161R) in a *taf1(ΔTAND)* strain and TBP(F182V). Bar graphs with each cluster are presented in the order listed above.
